# Assessment of Risk Factors for Musculoskeletal Pain Among University Staff Members

**DOI:** 10.3390/healthcare14010094

**Published:** 2025-12-31

**Authors:** Eman M. Mortada, Lujain F. Alshammari, Raseel S. AlShehri, Waad A. Asiri, Dima M. Alyousef

**Affiliations:** 1 Family and Community Medicine Department, College of Medicine, Princess Nourah bint Abdulrahman University, Riyadh 11671, Saudi Arabia; 2 Health Sciences Department, Health Sciences and Rehabilitation College, Princess Nourah bint Abdulrahman University, Riyadh 11671, Saudi Arabia; 442002236@pnu.edu.sa (L.F.A.); 442000272@pnu.edu.sa (R.S.A.); 442002424@pnu.edu.sa (W.A.A.); 442002920@pnu.edu.sa (D.M.A.)

**Keywords:** musculoskeletal pain, risk factors, staff members, office workers, work-related musculoskeletal disorders

## Abstract

**Background/Objectives**: Work-related musculoskeletal disorders (WMSDs) experienced by employees are the cause of significant issues and costs for companies. At PNU, understanding of the risk factors contributing to this pain is limited, impeding the development of effective solutions. To address this, it is important to examine various factors such as sociodemographics, ergonomics, psychology, and job satisfaction. By investigating these factors, PNU can create targeted interventions to improve worker health and reduce musculoskeletal pain. **Methods**: A cross-sectional study was conducted among 150 female staff members at the health colleges of Princess Nourah bint Abdulrahman University in Saudi Arabia, employing a multistage sampling technique. Data were collected from 20 December to 4 April 2024 using a standardized Google Forms questionnaire. Data analysis was performed using JMP software (version 14). **Results**: The results show a high prevalence of musculoskeletal pain among participants, with the analysis identifying several individual, ergonomic, and psychosocial risk factors that significantly correlated with reported pain, including prolonged sitting, poor posture, job stress, and low job satisfaction. **Conclusions**: Musculoskeletal disorders are prevalent among staff members, with ergonomic and psychosocial factors playing a significant role. Interventions targeting these risk factors are essential to improving occupational health and staff productivity.

## 1. Introduction

Musculoskeletal disorders (MSDs) represent a significant public health and occupational concern, ranking as the second most common cause of temporary or brief disabilities globally [1]. Among these, work-related musculoskeletal disorders (WMSDs) arise primarily from occupational exposure to triggers such as repetitive movements, prolonged static postures, and improper ergonomic conditions. These disorders affect muscles, tendons, ligaments, nerves, joints, and intervertebral disks, resulting in pain, discomfort, and reduced work capacity [2]. Numerous studies have highlighted the broad impact of WMSDs on healthcare costs, quality of life, employee productivity, absenteeism, and loss of work time [3].

Office workers are particularly vulnerable to WMSDs due to their prolonged sedentary behavior and repetitive tasks, such as extended computer use, which are known contributors to musculoskeletal strain [4]. Globally, MSDs constitute approximately 33% of all newly reported occupational diseases and are the leading cause of work-related sickness [5]. In Asian populations, the annual prevalence of WMSDs in at least one body region ranges from 40% to 95%, while in African countries, it ranges from 15% to 93.6% [6,7].

In Saudi Arabia, musculoskeletal symptoms have been reported by between 51% and 70% office workers, although comprehensive studies remain limited [8]. At Princess Nourah bint Abdulrahman University (PNU), the presence of musculoskeletal pain among staff poses a significant challenge to productivity, absenteeism, and overall well-being. Moreover, limited awareness of the contributing risk factors further impedes the development of effective workplace interventions [9,10].

Recent evidence also suggests that the shift toward technology-based work, including prolonged laptop use and mobile device dependency, has increased the ergonomic strain on office workers and contributes to higher rates of musculoskeletal discomfort [11,12]. Previous studies suggest that musculoskeletal pain is influenced not only by physical and ergonomic factors but also by psychosocial components such as job stress, workload, social support, and job satisfaction. However, evidence from female-dominated academic workplaces in Saudi Arabia remains scarce. Understanding these multidimensional risk factors within a single setting, a large women’s university, provides a unique occupational health perspective.

Therefore, the objective of this cross-sectional study is to identify the risk factors contributing to musculoskeletal pain among staff members at PNU. This study explores socio-demographic, ergonomic, psychological, and job satisfaction aspects to determine their role in musculoskeletal discomfort. It also evaluates the influence of stress levels, job demands, social support, and work–life balance as potential contributing factors. By analyzing data from a representative sample of employees, this study aims to provide evidence-based recommendations for preventive strategies and improved staff well-being at PNU and similar educational institutions. In addition, this study aims to assess the influence of stress levels, job demands, social support, and work–life balance as potential risk factors for musculoskeletal pain among PNU employees [7,8].

It is expected that important insights will be gained by investigating these factors, which in turn can help develop targeted interventions and practices to improve worker well-being and reduce musculoskeletal pain. Furthermore, the results of this study will contribute to existing knowledge on the topic by identifying specific risk factors associated with musculoskeletal discomfort among PNU workers [7,8]. The findings of this research will have significant practical implications for PNU and other similar educational institutions. The identification of specific risk factors will facilitate the implementation of evidence-based preventive measures, ergonomic interventions, and operational strategies aimed at improving the well-being of employees and reducing the incidence of musculoskeletal disorders. Ultimately, the study aims to promote worker well-being, increase job satisfaction, and minimize musculoskeletal pain in the workplace [7,8]. In summary, this cross-sectional study at PNU seeks to determine the risk factors for musculoskeletal pain among staff members, with the goal of implementing measures and practices that enhance worker well-being and minimize musculoskeletal pain at work. By establishing a relationship between musculoskeletal pain and the identified risk factors, this study aims to provide valuable insights that can aid in the development of targeted interventions and practices that will lead to improved health, productivity, and overall well-being among staff members at PNU and similar institutions.

## 2. Materials and Methods

### 2.1. Study Design

An analytical cross-sectional study design was used to determine the impact of sociodemographic, ergonomic, psychosocial, and job satisfaction factors on musculoskeletal pain levels.

### 2.2. Study Setting

The study was conducted at the six health colleges—the College of Medicine, the College of Dentistry, the College of Nursing, the College of Pharmacy, the Foundation Year for Health Colleges, and the College of Health and Rehabilitation Sciences—of Princess Nourah University, which is located in Riyadh, the capital of Saudi Arabia.

### 2.3. Study Duration

The study was conducted from October 2023 to June 2024, and the questionnaire was administered to participants during this period, between December 2023 and March 2024.

### 2.4. Study Population

#### 2.4.1. Inclusion Criteria

Female staff members working in office-based positions at different health colleges at Princess Nourah bint Abdulrahman University with at least one year of work experience were eligible to participate.

#### 2.4.2. Exclusion Criteria

Staff members who had worked for less than one year prior to data collection, those with a previous history of musculoskeletal disorders, and staff members aged below 24 years were excluded. This exclusion was applied to focus on work-related symptoms; however, it may have led to an underestimation of the true prevalence of musculoskeletal pain.

### 2.5. Sampling Procedures

#### 2.5.1. Sample Size

The sample of staff members for this study was calculated using the statistical formula for cross-sectional survey designs on the OpenEpi calculator (version 3). The anticipated population proportion (p) of the sample was estimated to be 50%, which was the safest choice since the sample size required is largest when P = 50% and no previous study on musculoskeletal pain (MSP) among staff members had been conducted at Princess Norah Bint Abdulrahman University. Other parameters for sample size calculation included a 95% CI and a marginal error of 5%. The sample calculated using the equation

$$n\;=\;\frac{\left[DEFF\;\times \;Np\left(1\;-\;p\right)\right]}{\left[{\frac{d^{2}}{z^{2}}}_{1-\alpha /2}\;\times \;(N\;-\;1)+p\;\times \;(1\;-\;p)\right]}\;\;\;\;\mathrm{comprised}\;\mathrm{of}\;195\;\mathrm{staff}\;\mathrm{members}.$$

Although the calculated sample size was 195, only 150 complete responses were obtained due to non-response and incomplete questionnaires. This shortfall may have reduced the statistical power of the study and should be considered when interpreting the regression results.

#### 2.5.2. Sampling Technique

A multistage sampling technique was employed. First, the health-related colleges at Princess Nourah bint Abdulrahman University were treated as strata. Second, the number of eligible female staff members in each college was obtained, and proportional allocation was applied according to the size of each college as revealed in the following Table 1.

Finally, the link to the online questionnaire was distributed to eligible staff members within each stratum through institutional communication channels, and those who met the inclusion criteria and provided informed consent were included as participants in the study.

### 2.6. Data Collection Tools

Data were collected using a structured, self-administered online questionnaire developed from validated instruments, such as the Dutch Musculoskeletal Questionnaire (DMQ) and ergonomic assessment tools, in addition to investigator-developed items addressing psychosocial factors and job satisfaction. The questionnaire consisted of the following four sections:

Section I covers sociodemographic details, academic background, and physical characteristics, and includes 31 items.

Section II is related to ergonomic factors (e.g., workstation setup, posture, and equipment) and compromises 27 items. The questions used to investigate the sociodemographic and ergonomic data were collected from the reviewed literature on potential risk factors for WRMSDs and were also derived from the standardized Dutch Musculoskeletal Questionnaire (DMQ).

Section III assesses psychosocial factors with mostly binary (yes/no) responses, except for 27 closed-ended questions, divided into five sections (mental tiredness at the end of the workday, shortage of personnel, being rested after a break, carrying out the same work all day, and becoming annoyed by others).

Section IV assesses job satisfaction: The questionnaire consisted of 91 multiple-choice, Likert-type, binary (“yes”/“no”) questions, and closed-ended questions. The questionnaire was made available in the English language and was distributed online using the online tool Google Forms. The results of the questionnaire were used to evaluate the risk of developing musculoskeletal pain among faculty members at Princess Nourah University using a standardized, self-administered survey instrument based on a reliable and validated questionnaire that had been used in previous research; both the Dutch musculoskeletal questionnaire and the Ergonomic questionnaire assessment were used. A pilot test of the questionnaire was conducted among some of the staff members at the colleges included in this study. The pilot test enabled the researchers to evaluate the validity and feasibility of the content, and the time required for the questionnaire’s completion. A few minor adjustments were applied to the questionnaire to improve the clarity and comprehensibility of some questions. Furthermore, Cronbach’s alpha was used to assess the instrument’s reliability, and a value of 0.71 was considered to reflect acceptable consistency.

#### Scoring System

All questionnaire responses were coded numerically prior to analysis. Most dichotomous (yes/no) items were originally coded as 1 = Yes and 2 = No. Items with multiple response categories were coded sequentially according to the order of responses provided in the questionnaire. For example, job satisfaction items with four response options were coded as 1 = good, 2 = reasonably good, 3 = not too bad, and 4 = bad. Continuous variables such as age, height, weight, hours of sitting per day, number of breaks, and minutes of rest were entered as reported by the participants.

A total of 19 negatively worded items across the ergonomic and psychosocial sections were reverse-coded to ensure that higher scores consistently reflected greater exposure to risk. Reverse coding was applied to items where a “Yes” response originally indicated a more favorable condition or lower risk (e.g., being rested after a break, having enough time to complete work on time, or having good back support). For these items, the original coding (1 = Yes, 2 = No) was transformed to (1 = No, 2 = Yes) prior to analysis. Musculoskeletal pain was treated as a binary outcome variable and analyzed as present/not present. Composite scores were not calculated; instead, individual variables were analyzed separately in the statistical models.

### 2.7. Statistical Analysis

The collected data were revised for completeness and consistency, coded, and entered into Microsoft Excel 2019. Then, they were exported to the JMP software package, version 14, for data analysis. All of the questions were scored, and scores of 1 or 2 were assigned to questions with a yes or no response. The Likert scale utilized in this study consisted of a single question, and the responses were assigned scores of 1, 2, 3, and 4, respectively. For the multiple-choice questions, a multiplicity scale was employed, with scores of 1, 2, 3, 4, and 5 assigned in sequential order. The findings are presented in the form of descriptive statistics, and frequency tables were created to show the distribution of responses for every variable. These tables will give an overview of the patterns and prevalence of musculoskeletal pain among staff members concerning job satisfaction, ergonomic, psychological, and sociodemographic variables. Multiple logistic regression and chi-square tests were conducted to assess the associations between musculoskeletal pain and the demographic variables, ergonomic variables, psychosocial variables, and job satisfaction variables. A significance threshold of 0.05 was used for the *p*-value.

### 2.8. Ethical Considerations

The study was approved by the Institutional Review Board of the Princess Norah University Research Center (IRB:24-0256). Informed consent was obtained electronically from participants before they began completing the online questionnaire, and they were made aware that they could withdraw from or leave the study at any point without feeling obligated to continue, and the objectives of the study were explained to them before they began answering the survey. The anonymity and confidentiality of all collected data were ensured.

## 3. Results

### 3.1. Descriptive Statistics

As shown in Table 2, the ages of the PNU staff members range between 24 and 50 years old. Most participants (51.33%) were aged between 24 and 30; the fewest (6.67%) were 50 years old and older. In terms of nationality, most staff members were Saudi (70.67%), with a lower proportion being non-Saudi (29.33%). In terms of income level, most participants (66.76%) were in the middle-income bracket, with the fewest (5.33%) being in the low-income bracket. In terms of teaching experience, most participants (46%) have between 11 and 15 years of teaching experience, followed by 38% who have between 6 and 10 years of experience; more than half (58.67%) have an academic load of between three and four courses, and the smallest category (11.33%) are those with four courses and above. Most participants do not engage in physical activity (76%). Regarding sleep, the highest percentage (72.67%) of participants get between 7 and 10 h of sleep per night, and the lowest (2%) get 11 h or more. In terms of BMI, 59.33% of the participants had a normal BMI, 33.33% were overweight, and 7.33% were obese. In terms of marital status, we found an equal percentage (38%) of divorced and married people, and 5.33% were widowed; most of them (42.67%) have 1–2 children, and 6% have 5 or more children.

### 3.2. Association Between Risk Factors and Musculoskeletal Pain

#### 3.2.1. Sociodemographic Characteristics and the Prevalence of MSP

Table 3 shows the association between the prevalence of MSP and sociodemographic factors. Marital status (*p* = 0.03), nationality (*p* = 0.006), education level (*p* = 0.01), and sleeping hours (*p* = 0.016) were significantly associated with MSP. Participants who slept fewer hours (3–6 h per night) reported a higher pain prevalence than those who slept for longer. Age, income, work experience, BMI, and physical activity were not significantly associated with MSP.

#### 3.2.2. Ergonomics Factors and the Prevalence of MSP

Table 4 reveals the association between the prevalence of MSP and ergonomic factors.

Participants who use a computer for ≥5 h per day reported a significantly higher prevalence of MSP compared with those who used a computer for fewer hours (*p* = 0.015). Multiple work-related postures were strongly associated with MSP. Prolonged standing (*p* = 0.0063) and prolonged sitting (*p* = 0.0144) were both linked to a higher prevalence of pain. Activities such as walking long distances (*p* = 0.0097), back or trunk bending (*p* = 0.0146), and repetitive hand or wrist movements (*p* = 0.0012) were also significantly associated with MSP. Maintaining an uncomfortable posture for extended periods showed the strongest association (*p* = 0.0003). In terms of equipment used, only headset use demonstrated a significant association with MSP (*p* = 0.0117), suggesting that specific ergonomic adjustments may influence pain outcomes depending on their implementation.

#### 3.2.3. Psychosocial Factors and the Prevalence of MSP

As shown in Table 5, two psychosocial variables demonstrated significant associations with MSP. Participants who could not choose the timing of their breaks (*p* = 0.0429) and those who frequently hurried to meet deadlines (*p* = 0.0046) reported higher levels of pain. Other psychosocial factors—such as the ability to choose work start/end times or the number of breaks taken per day—did not exhibit significant associations.

#### 3.2.4. Job Satisfaction and the Prevalence of MSP

Table 6 indicates no statistically significant associations between elements of job satisfaction (supervision, work atmosphere, support from colleagues, satisfaction with compensation, or perceived job suitability) and MSP (*p* > 0.05). Although many participants reported positive job satisfaction, this did not translate into differences in the prevalence of MSP.

**Figure 1:**
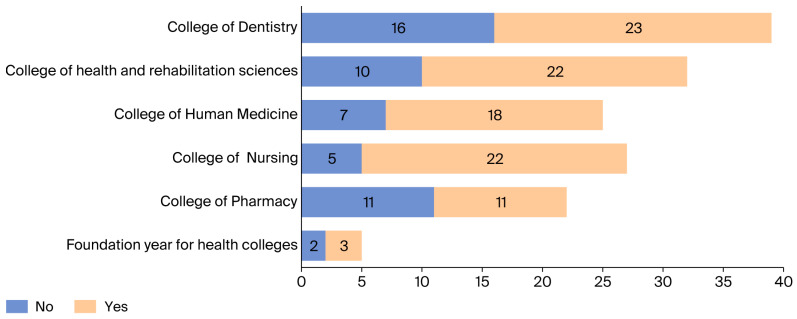
Discomfort and pain among staff members in different colleges over the last 12 months.

**Figure 2:**
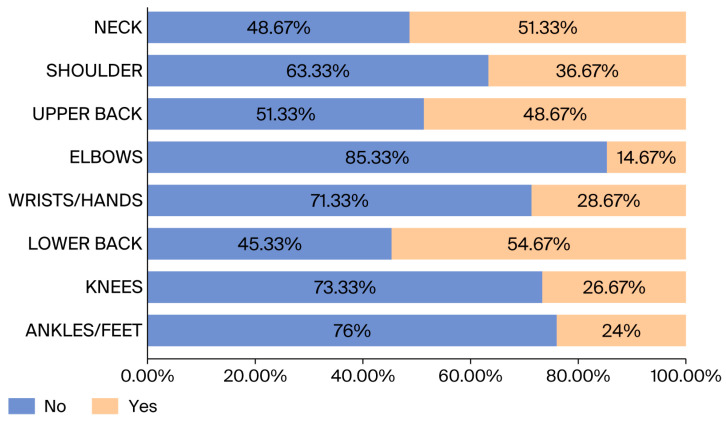
The prevalence of musculoskeletal pain among staff members in the past 12 months.

Figure 1 shows variation across colleges, with the College of Dentistry reporting the highest proportion of staff experiencing pain. In addition, As illustrated in Figure 2, the lower back was the most commonly affected region (54.67%), followed by the neck (51.33%) and upper back (48.67%). Pain in the elbow was reported least often (14.67%).

**Figure 3:**
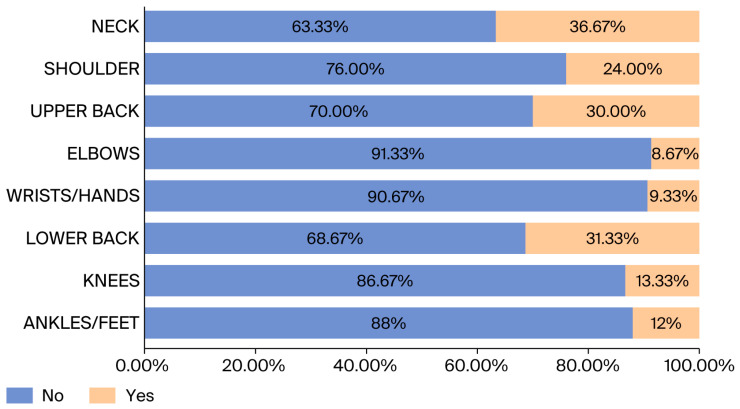
Prevalence of medication used for MSP among staff members.

Figure 3 demonstrates that medication use was most common for neck pain (36.67%), reflecting its severity and functional impact. Overall, 66% of the participants reported experiencing musculoskeletal pain within the past 12 months.

Figure 3 illustrates the use of medication for musculoskeletal pain by staff members. The most affected part of the body was the neck (36.67%), while the least affected was the elbow (8.67%).

Table 7 presents the results of the multivariable logistic regression analysis. Among the variables included in the model, prolonged maintenance of an uncomfortable posture was the only factor that remained statistically significant (OR = 0.70, 95% CI: 0.48–0.99, *p* = 0.048). This finding indicates an association between prolonged uncomfortable posture and musculoskeletal pain among the study participants.

## 4. Discussion

The prevalence of musculoskeletal pain (MSP) experienced by workers is a global issue that imposes significant burdens, such as absenteeism, lost productivity, and higher medical, disability, and workers’ compensation expenses [9,10]. Investigating different sociodemographic, ergonomic, psychological, and job satisfaction factors influencing MSP provides valuable knowledge that will aid in the development of focused interventions and procedures to enhance employee well-being and lessen the incidence of MSP. This study was conducted to assess the prevalence and associated factors of MSP among Princess Nourah University staff members in Riyadh, Saudi Arabia.

### 4.1. Prevalence of MSP

The prevalence of MSP in the past 12 months was 66%, 95% CI: 60.4% to 69.8%. This falls within the reported ranges among Asian staff 40% to 95%, employees in African nations (15% to 93.6%), and limited Saudi studies 51% to 70% [4,5,6,7,8,9,10,11,12,13,14]. This variation may be due to differences in data collection methods, sample sizes, and sampling techniques. For instance, the West Bengal study used face-to-face interviews and field visits, which may have introduced interviewer bias, while this study used an online survey, which is self-reported, enabling participants to recall any previous pain/discomfort they experienced. Additionally, there are methodological and participant characteristics differences between the Malaysian study and the Democratic Republic of Congo study. Monash University Malaysia used a large sample size (660), whereas this study included 150 participants. Moreover, the participants in the Democratic Republic of Congo study were gold mine workers, which makes it different to this study, which involved only staff members in a university setting.

### 4.2. Ergonomic Factors and Musculoskeletal Pain

A study conducted among secondary school Saudi female teachers found that musculoskeletal pain was significantly associated with work-related ergonomic factors, including prolonged standing, awkward postures, and repetitive tasks [14]. Meanwhile, this study showed no relationship between ergonomic conditions and MSP. Differences in findings may be due to varying sample sizes and the nature of the participants’ work.

In this study, lower back pain had the highest prevalence at 54.67%, followed by neck (51.33%) and upper back pain (48.67%). Interestingly, a separate study conducted among office workers at the University of Nigeria yielded similar results, where lower back pain affected 58.1%, wrist/hand pain 53%, and shoulder pain 50.2% of participants [15]. These findings suggest that lower back pain, wrist/hand pain, and shoulder pain are commonly observed among office workers. In the current study, a significant association (*p* < 0.05) was found between the number of hours per day spent using a computer and MSP, indicating that the relationship between these variables is statistically significant. This is considered a meaningful link between the duration of computer usage and the occurrence of MSP. Additionally, the study in Tampere, Finland, found no strong association between the duration of computer or mouse use and pain. This suggests that time spent using a computer or mouse may not be directly associated with pain [16]. It is also essential to recognize that variations in study design, participant demographics, and methodological approaches can substantially influence reported outcomes related to work-related musculoskeletal disorders [17]. Such methodological heterogeneity should be considered when interpreting findings across studies and comparing results between different occupational and geographic contexts.

### 4.3. Psychological Factors and Musculoskeletal Pain

In this study, the association between the prevalence of musculoskeletal pain (MSP) and psychosocial factors was measured. Similarly, a study conducted among office workers at the Iranian Gas Transmission Company used a cross-sectional design to explore the relationship between organizational and personal factors and the prevalence of musculoskeletal disorders (MSDs). The findings of that study revealed a significant association (*p* < 0.05) between MSP and two psychosocial factors: employees choosing when to take a break by themselves and hurrying to meet deadlines, as determined by chi-square tests. Likewise, an Iranian cross-sectional study reported statistically significant associations (*p* < 0.05) between musculoskeletal symptoms and multiple psychosocial, organizational, and individual factors among office workers, including job satisfaction, work demands, and work organization [18]. 

These findings emphasize the importance of considering a comprehensive approach that includes psychosocial, organizational, and individual aspects when addressing MSP in occupational settings, and they point to the need for further detailed research on these associations. The observed association between hurrying to meet deadlines and MSP in this study may be mediated through increased psychological stress pathways. High job demands and time pressure can lead to sustained sympathetic nervous system activation and increased muscle tension, particularly in the neck/shoulder region, even during low-load tasks. Similarly, the lack of autonomy in choosing when to take a break may prevent workers from taking timely micro-breaks to relieve accumulated physical strain and mental fatigue, thereby hindering physiological recovery and increasing the risk of developing musculoskeletal disorders [19].

### 4.4. Job Satisfaction and Musculoskeletal Pain

This study examined the relationship between job satisfaction and the prevalence of musculoskeletal pain (MSP). Among the 150 participants, 83.33% were satisfied with how they are supervised, while 7.33% were dissatisfied. However, no statistically significant association was found between MSP and job satisfaction. In contrast, a cross-sectional study among 210 male office workers at the Iranian Oil Company reported a higher prevalence of musculoskeletal disorders in the neck 45.70%, waist 41%, and knee 38.10%.

That study found a significant association between job satisfaction and musculoskeletal disorders affecting multiple body regions using similar measurement tools [11]. These findings highlight the need for preventive and ergonomic measures in workplaces to reduce musculoskeletal disorders and improve job satisfaction.

### 4.5. Implications and Future Directions

This study emphasizes the pressing need for ergonomic training and the provision of supportive equipment and psychosocial support for university staff [9,10]. Institutions should prioritize workstation assessments, encourage regular physical activity, and promote healthy work–rest cycles [19]. 

Future research should consider longitudinal designs to assess causality, include objective ergonomic assessments, and explore the role of psychosocial interventions [20]. Additionally, qualitative research could explore staff experiences and barriers to MSP prevention [10].

## 5. Conclusions

In conclusion, this research has provided valuable insights into the prevalence of musculoskeletal pain (MSP) among staff members at PNU. The study revealed that there is a moderate prevalence of MSP, with approximately 66% of the university’s staff experiencing this condition. Among the various types of MSP, lower back pain was found to be the most prevalent, affecting around 54.67% of the staff members, followed closely by neck pain, which was reported by 51.33% of the participants. In some of the usual working positions, working >5 h a day increases the experience of MSP among staff members. In addition, the regression model in this paper confirmed that hours spent sitting at a laptop/desktop computer was significantly associated with musculoskeletal pain.

## 6. Recommendations

Based on these specific findings, general improvements to working conditions are insufficient. University decision-makers should prioritize targeted ergonomic interventions aimed directly at reducing sedentary behavior and improving posture. Our recommendations include implementing mandatory short break policies to interrupt prolonged sitting cycles and providing practical ergonomic training focused on correcting uncomfortable posture habits. Furthermore, ensuring staff have access to appropriate adjustable ergonomic equipment (such as chairs and headsets) is crucial to mitigating these identified risks and improving staff well-being.

## 7. Limitation

This study has several limitations that should be considered when interpreting the findings. First, the cross-sectional design does not allow for causal inference between the identified factors and musculoskeletal pain. Second, data were collected using a self-administered questionnaire, which may be subject to recall bias and reporting bias. Third, an objective ergonomic assessment was not performed; therefore, workstation issues were based solely on self-reported information. Fourth, individuals with a previous history of musculoskeletal disorders were excluded, which may have led to an underestimation of the true prevalence of musculoskeletal pain. Fifth, the final sample size (N = 150) was smaller than the calculated required sample size, which may have reduced the statistical power of the regression model. In addition, multiple statistical comparisons were conducted without formal correction, which may have increased the risk of type I errors. Finally, the study was conducted at a single female university, which may limit the generalizability of the findings to other settings and to male populations.

## Figures and Tables

**Table 1:** The proportional allocation of the sample size from health**-related colleges in PNU**.

College of Health and Rehabilitation Science	91	91/394 × 195 = 45
College of Nursing	65	65/394 × 195 = 32
College of Pharmacy	78	78/394 × 195 = 39
College Dentistry	62	62/394 × 195 = 31
College of Medicine	90	90/394 × 195 = 44
Foundation Year for Health Colleges	8	8/394 × 195 = 4
Total	394	195

**Table 2:** Sociodemographic characteristics of staff members at PNU (N = 150).

Variables		Frequency (N)	Percent %
Age	24–30 years	77	51.33%
	31–35 years	16	10.67%
	36–40 years	14	9.33%
	41–45 years	19	12.67%
	46–50 years	14	9.33%
	>50 years	10	6.67%
Marital status	Single	28	18.67%
	Married	57	38%
	Divorced	57	38%
	Widowed	8	5.33
Income	High income level	42	28%
	Middle income level	100	66.67%
	Low income level	8	5.33%
Nationality	Saudi	106	70.67%
	Non-Saudi	44	29.33%
Level of education	Bachelor’s degree	4	2.67%
	Master’s degree	74	49.33%
	Ph.D.	72	48%
Work experience in years	1–5 years	12	8%
	6–10 years	57	38%
	11–15 years	69	46%
	>16 years	12	8%
BMI	Underweight	0	0%
	Healthy weight	88	58.67%
	Overweight	51	34%
	Obesity	11	7.33%
Number of children	0	18	12%
	1–2	64	42.67%
	3–4	59	39.33%
	>5	9	6%
Structured physical activity	Yes	36	24%
	No	114	76%
Sleeping hours	3–6 h	38	25.33%
	7–10 h	109	72.67%
	11 or more	3	2%

**Table 3:** Associations between sociodemographic characteristics and the prevalence of MSP among staff members.

Variables		Total N = 150	History of MSP in Past 12 Months		Chi-Square	*p*-Value
			Yes	No		
Age	24–30 years	77 (51.33%)	25 (16.67%)	52 (34.67%)	11.661	0.04 *
	31–35 years	16 (10.67%)	5 (3.33%)	11 (7.33%)		
	36–40 years	14 (9.33%)	5 (3.33%)	9 (6%)		
	41–45 years	19 (12.67%)	10 (6.67%)	9 (6%)		
	46–50 years	14 (9.33%)	6 (4%)	8 (5.33%)		
	>50 years	10 (6.67%)	4 (2.67%)	6 (4%)		
Marital status	Single	28 (18.67%)	8 (5.33%)	20 (13.33%)	8.859	0.03 *
	Married	57 (38%)	24 (16%)	33 (22%)		
	Divorced	57 (38%)	21 (14%)	36 (24%)		
	Widowed	8 (5.33%)	2 (1.33%)	6 (4%)		
Income	High income level	42 (28%)	30 (20%)	12 (8%)	1.241	0.54
	Middle income level	100 (66.67%)	63 (42%)	37 (24.67%)		
	Low income level	8 (5.33%)	6 (4%)	2 (1.33%)		
Nationality	Saudi	106 (70.67%)	62 (41.33%)	44 (29.33%)	12.591	0.006 *
	Non-Saudi	44 (29.33%)	37 (24.67%)	7 (4.67%)		
Level of education	Bachelor’s degree	4 (2.67%)	1 (0.67%)	3 (2%)	8.513	0.01 *
	Master’s degree	74 (49.33%)	43 (28.67%)	31 (20.67%)		
	Ph.D.	72 (48%)	55 (36.67%)	17 (11.33%)		
Work experience in year	1–5 years	12 (8%)	10 (6.67%)	2 (1.33%)	2.771	0.44
	6–10 years	57 (38%)	34 (22.67%)	23 (15.33%)		
	11–15 years	69 (46%)	47 (31.33%)	22 (14.67%)		
	>16 years	12 (8%)	8 (5.33%)	4 (2.67%)		
BMI	Underweight	0 (0%)	0 (0%)	0 (0%)	1.027	0.59
	Healthy weight	88 (58.67%)	60 (40%)	28 (18.67%)		
	Overweight	51 (34%)	31 (20.67%)	20 (13.33%)		
	Obesity	11 (7.33%)	8 (5.33%)	3 (2%)		
Structured physical activity	Yes	36 (24%)	27 (18%)	9 (6%)	1.710	0.19
	No	114 (76%)	72 (48%)	42 (28%)		
Sleeping hours	3–6 h	38 (25.33%)	31 (20.66%)	7 (4.67%)	5.85	0.016 *
	7–10 h	109 (72.67%)	66 (44%)	43 (28.67%)		
	11 and more	3 (2%)	2 (1.33%)	1 (0.67%)		

*p*-value reported by chi-square test. * *p*-value < 0.05.

**Table 4:** Associations between ergonomic factors and the prevalence of MSP among staff members.

Variable		Total	Prevalence of MSP in Past 12 Months		Chi-Square	*p*-Value
			Yes	No		
Using computer (hours per day)	<5 h	113 (75.33%)	42 (21.01%)	71 (36.45%)	5.92	0.0150 *
	> or = 5	37 (24.67%)	13 (12.33%)	24 (30.21%)		
Equipment	Ergonomic chair	87 (58%)	58 (38.67%)	29 (19.33%)	0.041	0.84
	Keyboard	65 (43.33%)	48 (32%)	17 (11.33%)	3.147	0.08
	Mouse	72 (48%)	46 (30.67%)	26 (17.33%)	0.275	0.6
	Headset	45 (30%)	23 (15.33%)	22 (14.67%)	6.351	0.01 *
	Desk	72 (48%)	52 (34.67%)	20 (13.33%)	2.389	0.12
Type of computer	Laptop	93 (62%)	31 (20.67%)	62 (41.33%)	3.512	0.17
	Desktop	35 (23.33%)	11 (7.33%)	24 (16%)		
	Both	22 (14.67%)	13 (8.67%)	9 (6%)		
Usual working position	Standing for long periods	58 (38.67%)	46 (30.67%)	12 (8%)	7.466	0.006 *
	Sitting for long periods	49 (32.67%)	39 (26%)	10 (6.67%)	5.991	0.01 *
	Lifting heavy objects	18 (12%)	13 (8.67%)	5 (3.33%)	0.353	0.55
	Pushing carts or heavy objects	13 (8.67%)	6 (4%)	7 (4.67%)	2.498	0.11
	Walking long distances	33 (22%)	28 (18.67%)	5 (3.33%)	6.698	0.019 *
	Back/trunk rotation	23 (15.33%)	19 (12.67%)	4 (2.67%)	3.339	0.07
	Back/trunk bending	28 (18.67%)	24 (16%)	4 (2.67%)	5.963	0.01 *
	Repetitive hand/wrist movement	39 (26%)	34 (22.67%)	5 (3.33%)	10.535	0.001 *
	Working with and/or operating large machines	16 (10.67%)	11 (7.33%)	5 (3.33%)	0.060	0.81
	Working with and/or operating machines that produce vibration	13 (8.67%)	7 (4.67%)	6 (4%)	0.937	0.33
	Maintaining an uncomfortable posture for long periods of times	50 (33.33%)	43 (28.67%)	7 (4.67%)	13.369	0.0003 *

*p*-value reported by chi-square test. * *p*-value < 0.05.

**Table 5:** Associations between psychosocial factors and the prevalence of MSP among staff members.

Variable		Total	Prevalence of MSP in Past 12 Months		Chi-Square	*p*-Value
			Yes	No		
Choosing the start and end of a working day	Yes	67 (44.67%)	41 (27.33%)	58 (38.67%)	1.246	0.26
	No	83 (55.33%)	26 (17.33%)	25 (16.67%)		
Choosing when to take a break (other than a prayer break)	Yes	58 (38.67%)	44 (29.33%)	55 (36.67%)	4.099	0.04 *
	No	92 (61.33%)	14 (9.33%)	37 (24.67%)		
Breaks per day	From 0 to 2 breaks	125 (66.8%)	43 (22.14%)	82 (44.66%)	0.04	0.85
	From 3 to 6 breaks	25 (33.2)	12 (15.81%)	13 (17.39%)		
Hurrying to be ready on time	Yes	80 (53.33%)	61 (40.67%)	19 (12.67%)	8.026	0.0046 *
	No	70 (46.67%)	38 (25.33%)	32 (21.33%)		

*p*-value reported by chi-square test. * *p*-value < 0.05.

**Table 6:** Associations between job satisfaction and the prevalence of MSP among staff members.

Variable		Total N = 150	Prevalence of MSP in Past 12 Months		Chi-Square	*p*-Value
			Yes	No		
Supervision	Yes	125 (83.33%)	81 (54%)	44 (29.33%)	0.481	0.49
	No	25 (16.67%)	18 (12%)	7 (4.67%)		
Atmosphere	Yes	78 (52%)	54 (36%)	24 (16%)	0.756	0.38
	No	72 (48%)	45 (30%)	27 (18%)		
Support from others	Yes	96 (64%)	65 (43.33%)	31 (20.67%)	0.347	0.56
	No	54 (36%)	34 (22.67%)	20 (13.33%)		
Appropriateness of compensation	Yes	98 (65.33%)	63 (42%)	35 (23.33%)	0.370	0.54
	No	52 (34.67%)	36 (24%)	16 (10.67%)		
Work suitability	Well	56 (37.33%)	37 (24.67%)	19 (12.67%)	1.253	0.74
	Reasonably good	49 (32.67%)	34 (22.67%)	15 (10%)		
	Not too bad	34 (22.67%)	20 (13.33%)	14 (9.33%)		
	Bad	11 (7.33%)	8 (5.33%)	3 (2%)		

**Table 7:** Multiple logistic regression of factors associated with musculoskeletal pain in the past 12 months (N = 150).

Variable	OR	CL 95%		*p*-Value
		Min	Max	
Years of teaching experience [6–10 years]	0.15	0.63	2.10	0.65
How many hours per day do you spend sitting at your desk/workstation using your laptop/desktop?	0.96	0.91	1.08	0.23
Maintaining an uncomfortable posture for long periods	0.70	0.48	0.99	0.048 *
Have you ever used or are currently using any ergonomic equipment or tools at work? [Headset]	0.78	0.54	1.13	0.18

* *p*-value < 0.05.

## Data Availability

The data presented in this study are available on request from the corresponding author due to privacy reasons.

## Abbreviations

The following abbreviations are used in this manuscript:

WMSDs	Work-related musculoskeletal disorders
MSDs	Musculoskeletal disorders
MSP	Musculoskeletal Pain
PNU	Princess Nourah bint Abdulrahman University
